# A combined pre-clinical meta-analysis and randomized confirmatory trial approach to improve data validity for therapeutic target validation

**DOI:** 10.1038/srep13428

**Published:** 2015-08-27

**Authors:** Pamela WM. Kleikers, Carlijn Hooijmans, Eva Göb, Friederike Langhauser, Sarah SJ. Rewell, Kim Radermacher, Merel Ritskes-Hoitinga, David W. Howells, Christoph Kleinschnitz, Harald HHW Schmidt

**Affiliations:** 1 Department of Pharmacology, CARIM, Faculty of Health, Medicine and Life Sciences, Maastricht University, The Netherlands; 2 SYRCLE at Central Animal Laboratory, Radboud University Medical Centre, Nijmegen, The Netherlands; 3 Neurologische Klinik und Poliklinik der Universitätsklinik Würzburg, Würzburg, Germany; 4 Florey Institute of Neuroscience and Mental Health, Austin Health, Melbourne, Victoria, Australia

## Abstract

Biomedical research suffers from a dramatically poor translational success. For example, in ischemic stroke, a condition with a high medical need, over a thousand experimental drug targets were unsuccessful. Here, we adopt methods from clinical research for a late-stage pre-clinical meta-analysis (MA) and randomized confirmatory trial (pRCT) approach. A profound body of literature suggests NOX2 to be a major therapeutic target in stroke. Systematic review and MA of all available NOX2^-/y^ studies revealed a positive publication bias and lack of statistical power to detect a relevant reduction in infarct size. A fully powered multi-center pRCT rejects NOX2 as a target to improve neurofunctional outcomes or achieve a translationally relevant infarct size reduction. Thus stringent statistical thresholds, reporting negative data and a MA-pRCT approach can ensure biomedical data validity and overcome risks of bias.

Medical innovation is impacted by major quality and reproducibility issues of basic science data^1^,^2^,^3^ that then impact on further clinical development^4^,^5^. These issues are on top of a general notion that animal models may be less predictive than we thought^6^,^7^. It has been shown that this is partly a consequence of suboptimal study design^8^ and analysis^9^, and poor reporting^10^,^11^,^12^.

In stroke and its preclinical research, the situation is particularly alarming^13^,^14^. Stroke is the third cause of death and number one reason for chronic disability. Despite this high medical need only one drug (recombinant tissue plasminogen activator) is registered to treat ischemic stroke; however, it has limited efficacy^15^ and over 30 contraindications, so that 85% of all stroke patients remain without any acute drug treatment. This single “successful” stroke drug development stands in sharp contrast to 1,026 failed experimental stroke targets^14^,^16^, despite several roundtable recommendations to improve the quality of preclinical stroke research^17^,^18^ including advanced study designs^19^. Consequently, industry scientists have left target discovery and drug development for stroke almost completely non-investigated. Recently, this has led to proposals that preclinical research needs to improve in quality by adopting elements of clinical research, including multi-center studies, randomization, blinding and a priori power calculation for relevant outcomes^17^,^20^,^21^.

With respect to relevant pre-clinical outcomes, a shift is necessary from surrogates such as infarct size to measurement of neurological function; with respect to the therapeutic approach, patients need a shift from vascular re-canalization to adding on neuroprotection^22^. Oxidative stress or the occurrence of reactive oxygen species (ROS) in increased amounts, in unphysiological places or with unphysiological chemistry, has been suggested to play a major role in neurodegeneration upon ischemic stroke^23^,^24^,^25^. Even under conditions of ischemia^26^, ROS have both protective and deleterious effects^27^, which explains why global anti-oxidant therapy has failed^28^,^29^,^30^. A more promising approach is to target oxidative stress in a manner that leaves essential, physiological ROS formation untouched and inhibits only disease-relevant enzymatic sources^30^,^31^,^32^. As such a source of ROS, NADPH oxidases (NOX) stand out as they represent the only enzyme family that has ROS formation as its only known function. Most prominently NOX2^33^ has been suggested to be a prime target in stroke^34^, whilst our own previous observations^24^ profoundly disagreed. Nine other publications (using a total of 159 animals), however, investigated NOX2^-/y^ mice to conclude a major contribution of this enzyme to infarct size by up to 60%^23^,^35^,^36^,^37^,^38^,^39^,^40^,^41^,^42^. As a point of concern, NOX2 is known to play a major role in innate immunity and its deletion causes severe immune deficiencies, in particular with common comorbidities such as diabetes mellitus^43^. Because of these risks, the benefit of inhibiting NOX2 in stroke would need to be validated beyond doubt before entering a discovery program or clinical trials.

Adoption of large-scale collaborative research has been suggested as a means to improve the success rate and validity of such pre-clinical target validation. Clearly this is not feasible for every exploratory pre-clinical trial. However, in order to make a valid target statement, we here learn from the successes of clinical research and implement for the first time a recent suggestion^21^ to conduct pharmacological target validation research as pre-clinical randomized confirmatory trials (pRCTs). We also combine this with a systematic review (SR) and meta-analysis (MA) that is then re-run after the pRCT. The outcomes of the SR-MA-pRCT-MA approach have implications for stroke research and late-stage preclinical biomedical research in general.

## Methods

### Animals

NOX2 deficient mice (NOX2 KO, stock #002365) from C57Bl/6J background and corresponding age-matched C57Bl/6J control mice (stock #000664) with an SPF health status were purchased from Jackson Laboratories (Bar Harbor, ME, USA). In a previous study^24^ we already tested young male (6–8 weeks, 20–25 grams) mice. We therefore extended our inclusion criteria by using also female (8–10 weeks, 18–21 grams) and older (18–20 weeks, 26–31 grams) mice. All experiments were approved by the local animal ethics committees of Maastricht (DEC 2011-106) and Würzburg (69/08). Animals were socially housed in IVC cages under controlled conditions (22 °C, 55–65% humidity, 12 h light-dark cycle, in type II IVC macrolon cages up to 3 mice in Würzburg, up to 4 males and 5 females in Maastricht; type III, up to 10 in Würzburg), and were allowed free access to water and standard laboratory chow (Maastricht, R/M-H, ssniff, Soest, Germany; Würzburg, Altromin standard diet, Altromin Spezialfutter GmbH & Co. KG, Lage, Germany).

### Systematic review

The present review was based on published results of animal studies on the role of NADPH oxidase 1 and/or 2 in experimental ischemic stroke. PubMed and EMBASE were searched for original papers and conference abstracts concerning the effects of NADPH oxidase 1 and/or 2 on experimental stroke until October 23, 2013. The search strategy involved the following 3 search components: ischemic stroke, NADPH oxidase 1 and/or 2 and animals (for the complete search strategy, see Supplementary Table 1). For detecting animal studies, search filters developed by SYRCLE were used^44^,^45^. No language restriction was used. Our search strategy identified 562 records in PubMed and 812 records in EMBASE. After removal of duplicates, a total of 1089 records were screened a first time based on title and abstract, excluding non-in-vivo papers, papers not using ischemic stroke and papers using an unspecific inhibitor of NOX or combining an inhibitor with other therapies. 25 articles were included for full text screening, of which 22 addressed NOX2 and 4 articles addressed NOX1. Two independent researchers (PWMK and SSJR) screened all titles and abstracts for the inclusion criteria. Studies were included if they 1) investigated the role of NADPH oxidase 1 and/or 2 on the infarct size and neurological scoring after experimental focal ischemic stroke using either genetic or specific pharmacological inhibition of these NOX isoforms; 2) were performed in animals *in vivo*; 3) resulted in an original full paper or conference abstract which presented unique data. Papers were excluded when unspecific NOX inhibitors such as apocynin^46^,^47^ were used or when NOX inhibition was combined with other drugs/therapies. The in– and exclusion criteria and methods of analysis were specified in advance and documented in a protocol.

After full text assessment, thirteen papers for the NOX2 study (using in total 162 WT and 171 KO mice) and four papers for the NOX1 study (using in total 69 WT and 141 KO mice), and 16 rats (8 with and 8 without siRNA) were included (Supplementary Fig. 1) for qualitative and quantitative analyses^23^,^24^,^36^,^37^,^38^,^39^,^40^,^41^,^42^,^48^,^49^,^50^,^51^,^52^,^53^.

### Study characteristics

From the included studies, bibliographic data such as authors, year of publication, journal of publication and language were registered. Study characteristics concerning study design were extracted and summarized in Supplementary Table 2: species, strain (including genetic KO), gender, age and weight of the animals used; type of anesthesia; method and duration of ischemia; duration of reperfusion (timing of outcome measurements), type of inhibitor used; method of culling; method of infarct size measurement and neurological outcome assessment; (reason for) dropouts and mortality. All studies except one, used NOX KO mice as experimental animals, with one study using both genders, the rest only males. One study used rats treated with siRNA. All studies used the middle cerebral artery (MCA) occlusion model, sixteen studies occluded transiently allowing reperfusion afterwards, and two studies occluded the MCA permanently (one study used both permanent and transient ischemia). The duration of the ischemia and the reperfusion varied greatly among studies (5 to 120 minutes). Three different methods of infarct size measurement and three different scoring systems for the neurological assessment were used. All retrieved data sets could be taken into account for the meta-analysis regarding infarct size. Neurological scoring was measured in seven out of thirteen NOX2 papers and three out of four NOX1 studies.

### Assessment of methodological quality and risk of bias

Study quality and risk of bias in the included studies was independently assessed by two reviewers (PWMK and CH), using a predefined 9-point rating system (based on^54^) (see Supplementary Table 3 and legend for details). Seven items were assessed to study risk of bias. A “yes” judgment indicates a low risk of bias; a “no” judgment indicates high risk of bias; the judgment was “unclear” if insufficient details had been reported to assess the risk of bias properly. The possible presence of selection bias (items 1, 2 and 4) detection bias (items 6, 7 and 8) performance bias (item 5) and attrition bias (items 9) were judged. Because of poor reporting of essential details in animal studies, we also included two reporting items: we assessed whether it had been reported if the experiments were randomized or blinded at any level (item 1 and 3). Disagreements were solved by discussion.

### Meta-analysis

Infarct size and neurological outcome were included in the meta-analysis. Data were extracted if raw data or group averages, standard deviation (SD) or standard error (SE), and number of animals per group (n) were reported, or could be recalculated. All authors were contacted to contribute their original data to the meta-analysis. From two publications, no response from authors was obtained. For one publication, authors could not recollect their original data. In these cases, data were extracted from the text, or if presented only graphically, measured using a universal on-screen digitizer (Universal Desktop Ruler). CAMARADES consortium (http://www.dcn.ed.ac.uk/camarades//default.htm) suggests the use of normalized mean difference (NMD), which requires correcting for sham values. However, of all thirteen included NOX2 papers, only three reported the use of shams, but not for all groups and all outcome parameters^35^,^38^,^42^. None of the others mention any sham animals. Therefore, we applied the standardized mean difference (SMD) which is also regularly used in clinical meta-analyses^55^ for both the outcome measure ‘infarct size’ and ‘neurological score’. An SMD expresses the difference between the groups relative to the standard deviation. Calculation of mean differences^56^,^57^,^58^ was not possible because of the heterogeneity in study designs (i.e. species) and the variety of scales used to determine the outcomes. The studies of NOX1 and NOX2 were analyzed separately. In case different measures of neurobehavioral outcomes were reported from the same cohort of animals we pooled the individual effect sizes and used this pooled estimate in the overall meta-analysis. Despite anticipated heterogeneity, the individual SMDs were pooled whenever possible (starting from two studies or more) to obtain an overall SMD and 95% confidence interval.

To account for anticipated heterogeneity, we used the random effects model in which some heterogeneity beyond sampling errors is allowed. In order to assess the robustness of our findings and in an attempt to explain observed study heterogeneity, we performed a sensitivity analysis and we investigated the effects of excluding the study with permanent ischemia. Meta-analysis was performed using Comprehensive Meta Analysis (CMA version 2.0). Forest plots were used to display the mean overall effect sizes. Data are expressed as SMD with 95% confidence intervals. For the outcome measure infarct size, we assessed the possibility of publication bias by visually evaluating the possible asymmetry in funnel plots^59^. Using the trim and fill analyses an adjusted intervention effect was calculated^60^.

### pRCT

The preclinical Randomized, Confirmatory (and blinded) animal Trial was performed in parallel at Maastricht University (The Netherlands) and at the University of Würzburg (Germany). All animals studies were done in accordance with the approved national animal experimental guidelines and were approved by the local animal ethics committees. The objective of the study was to compare the extent of neurological damage after stroke in mice with or without NOX2 gene deletion. At each study site, surgery and follow-up measurements were performed blinded and animals were operated randomly according to an online randomization tool (www.randomizer.org). For a power of 80%, based on a minimal effect on infarct size of −40% and an SD of 30%, the required animal numbers were at least n = 10 per study arm. Transient middle cerebral artery occlusion (tMCAO) was performed with an intraluminal filament method as described by Kleinschnitz *et al.*^24^. After 60 minutes of ischemia, the filament was withdrawn and reperfusion established. Twenty-four hours after induction of the ischemia, mice were scored for neurological and motor function. Infarct size was determined using 2, 3, 5-triphenyltetrazolium chloride (TTC) staining. For a more detailed description, see the Supplementary Information Methods.

### Power analysis

We conducted a *post hoc* analysis of power in all earlier published studies in Nox2^-/y^ mice and stroke. Twelve studies and our own new data were analyzed for their power to detect a difference of 40% in infarct size. This threshold of 40% difference was based on post-hoc analysis of failed clinical trials where preclinical studies showed a 30–40% difference^29^,^61^,^62^. Power was calculated using Russ Lenth’s power software, an alpha of 0.05, an effect of 0.4 and a pooled variance [(Lenth, R.V 2006-9, java Applets for Power and Sample Size [Computer Software], Retrieved 02-17-2014, from http://www.stat.uiowa.edu/~rlenth/Power)]. The pooled variance was calculated for 4 different groups according to ischemic and reperfusion time: a) long reperfusion time (72 h), b) short reperfusion time (24 h) after short ischemia (30 min), c) short reperfusion time (24 h) after medium ischemia (60-75 min), and d) short reperfusion time (24 h) after long ischemia (90-120 min). From each individual study, the coefficient of variation was calculated for both KO and WT values. Taking into account all these values, the pooled variance for each group was calculated according to formula (1) here with n the size of the group and CV the coefficient of variation (SD/Mean) of the group.


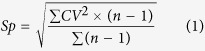


### Statistics

Infarct volume data are expressed as mean ± SEM. Statistical differences between mean values were determined by Student’s two-tailed t test, using the GraphPad Prism 5.0 software package. Neurological scores were expressed as median. For discrete variables (behavior and motor function scores), the Mann-Whitney U-test was used. A value of P < 0.05 was considered to be statistically significant. Power calculations were performed by using Russ Lenth’s power and sample size software.

## Results

### Systematic review and meta-analysis suggest a role of NOX2 in stroke

**Figure 1:**
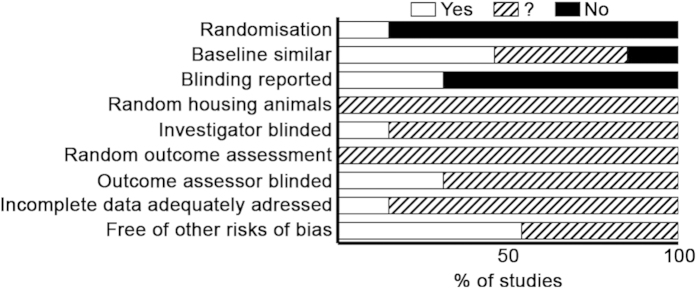
Risk of bias and reporting quality of NOX2 studies, averaged per item. Items, i.e. risks of bias are listed on the left. Open bars indicate a low risk of bias; closed, a high risk; hatched bars, an unclear risk. Items 1 and 3 scored reporting.

**Figure 2:**
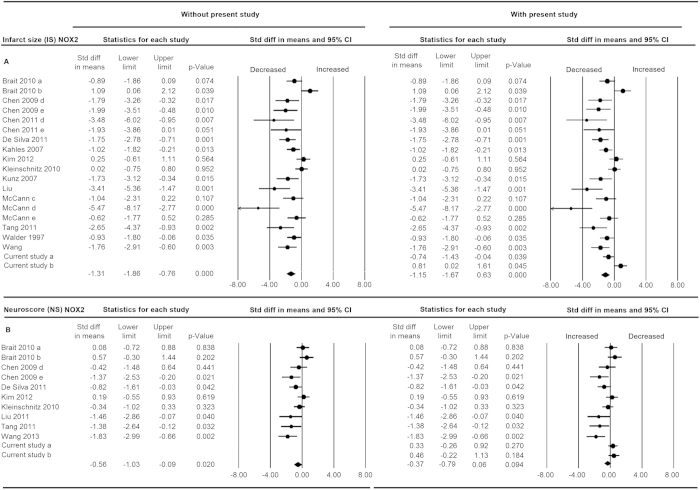
Meta-analysis of the overall effect of NOX2 on infarct size and neurological score in experimental stroke. Studies included are shown on the left and analyzed in two forest plots, either without (left section) or with (right section) the data of the here presented randomized, confirmatory, blinded study. Subgroups within one study are depicted separately with the following coding: a, female gender; b, male gender; c, short ischemic time; d, medium ischemic time; e, long ischemic time. The upper half a, contains data for the effect of NOX2 on infarct size (IS); the lower half b, on neurological score (NS). Displayed are the standardized mean difference (SMD), 95% confidence intervals and relative weight of the individual studies. The diamond indicates the global SMD and its 95% confidence interval.

In preparation for a risk of bias and statistical power analysis on pre-clinical animal studies on NOX2 in experimental stroke, we first conducted a systematic review^63^ (for search strategy see Supplementary Table 1, Supplementary Fig. 1) followed by a meta-analysis (for study characteristics see Supplementary Table 2)^23^,^24^,^35^,^36^,^37^,^38^,^39^,^40^,^41^,^42^,^53^,^64^,^65^. Heterogeneity was relatively high (I^2^ = 73%) as expected given the large variations in study designs and methodological quality (Supplementary Table 2, Fig. 1). All studies on NOX2 in stroke were conducted in mice. One study created a permanent occlusion^48^ and all studies but six^23^,^37^,^39^,^41^,^53^,^65^ studied neurological outcome 24 h after ischemia. Indeed, we found that the reported infarct size for NOX2 KO vs. NOX2 WT infarct size was significantly smaller (Fig. 2a). NOX2 KO mice also showed a small but significant decreased neurological score compared to WT mice (Fig. 2b), which implicates an improved neurological function (SMD −0.67 [−1,17; −0,16]; n = 9, p = 0.010). Calculation of mean differences^56^,^57^,^58^ was not possible because of the heterogeneity in study designs (i.e. species) and the variety of scales used to determine the outcomes (see Methods).

### Most studies on NOX2 in stroke were insufficiently powered

We then analyzed whether those studies that were included in the systematic review were sufficiently powered to detect an effect size of 40%, which is considered a minimum to be subsequently translationally relevant (see Methods). Since especially in small studies the observed variance is not a precise estimate of the true variance, we computed pooled variances. For four different groups (short ischemia time, medium ischemia time, long ischemia time and long reperfusion time), we found these calculated pooled variances to be 0.44, 0.60, 0.29 and 0.43, respectively. With these and the number of animals described in each study, we calculated the power to detect a difference of at least 40% (Table 1). Most notably, none of the studies reached a sufficient level of power (1-ß ≥ 0.08), with the exception of our own earlier study (1-ß = 0.95), which had shown no effect for NOX2^24^.

### Poor reporting and risk of bias in studies on NOX2 in stroke

To assess the quality of the included papers, we conducted a risk of bias analysis. However, as a result of poor reporting in most animal studies, we also assessed a few reporting criteria (item 1 and 3). Figure 1 shows this risk of bias results for the NOX2 studies. Only a single study reported randomization. Also, in only 15% of the studies it was clear that at baseline groups were similar with respect to age, weight, and supplier. None of the papers mentioned whether or not they housed the animals randomly and used a random order to assess the outcomes. Only four papers reported blinding at any level, for two of these four studies, both the treatment and assessment, for the other two only the outcome assessment was blinded. Overall, the risk of bias analysis showed that reporting essential details of the animal stroke studies is poor and there seems to be a substantial risk of bias.

### Publication bias leads to an overestimation of the NOX2 effect size in stroke

**Figure 3:**
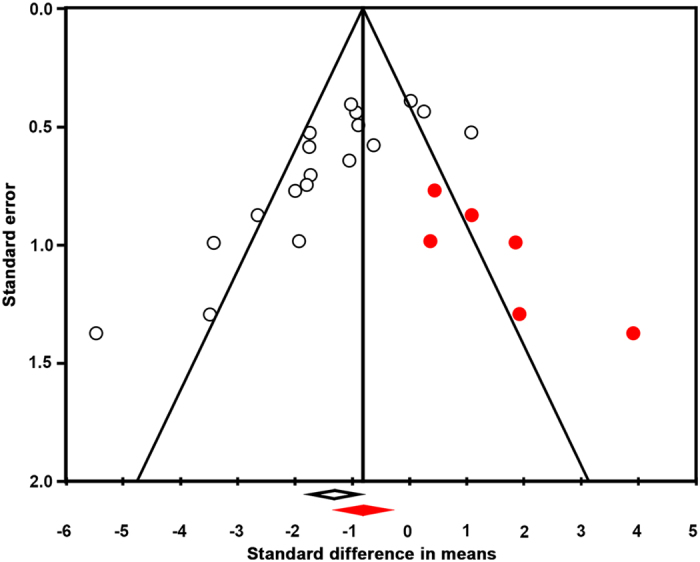
Funnel plot asymmetry suggesting the presence of publication bias and an overestimation of the overall effect size of NOX2 in stroke. The Y-axis represents precision; the X-axis effect size of individual studies. The funnel plot is based on the fact that precision in estimating the underlying treatment effect will increase as the sample size of component studies increases. Using the trim and fill analyses the intervention effect is adjusted for possible missing studies (filled red symbols) amongst published data (open symbols). The asymmetry suggests that studies showing larger infarcts in NOX2 KO mice in experimental stroke are missing. These would otherwise shift the mean (open diamond) towards a smaller or no overall effect of NOX2 (closed diamond).

In addition to insufficient power, publication bias has been shown to influence preclinical study results. To identify a possible publication bias for the outcome ‘infarct size’, we created a funnel plot^10^,^60^. Figure 3 shows that it is likely that several small studies reporting larger infarcts in NOX2 KO mice are missing from the literature. This may indicate an overestimation of the overall effect (SMD −0,66 [−1,20; −0,14]) as calculated in the meta- analysis and questions the significance of NOX2. Moreover, reporting of essential details of the animal stroke studies was poor and there was a substantial risk of bias (Fig. 1). Only 31% of the studies reported on blinding of the outcome measures. In addition, just 15% of the studies described whether they randomized the allocation of the animals to the various groups. None of the papers described the method for randomization. Blinding and randomization are key quality measures of experimental design of intervention studies, and are known to cause bias^54^.

### A randomized, confirmatory, blinded, and fully powered pre-clinical trial excludes a relevant role for NOX2 in stroke

**Figure 4:**
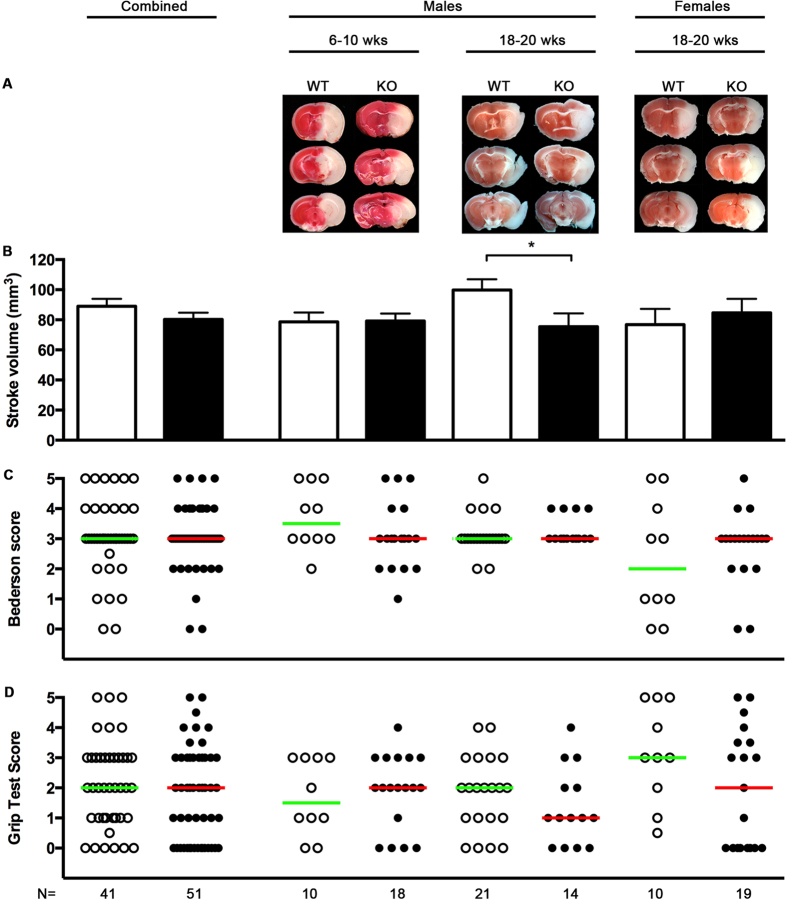
Infarct size and neurological function in NOX2 and WT mice. Mice of both genders and between 6–20 weeks old were subjected to tMCAO. (**A**) TTC stainings of three sequential coronal brain sections on day 1 after tMCAO that were representative for each subgroup: young (6–10 week old), adult (18–20 week old) male, and adult female NOX2 KO and WT mice. The TTC staining colors viable tissue red, while infarcted tissue stays white. (**B**) Bar graphs of mean infarct volumes ± SEM from WT (open bar) and NOX2 KO mice (closed bars). (**C**) Scatter plot and median of neurodeficit Bederson scores on day 1 after tMCAO, ranging from 0 (normal) to 5 (severe), from WT (open symbols) and NOX2 KO mice (closed symbols). (**D**) Scatter plot and median of grip test scores, ranging from 0 (severe deficit) to 5 (normal) from WT (open symbols) and NOX2 KO mice (closed symbols). The asterisk, *, indicates statistical significance (P < 0.05, t-test) for the infarct size in the subgroup adult male NOX2 versus WT; however, this group was insufficiently powered to allow the detection of a difference. Combined analysis did not show a significant difference for any of the parameters.

The literature on NOX2 in stroke fulfilled all criteria to justify conducting the first pre-clinical, randomized, confirmatory trial, powered for a minimally relevant effect of 40% reduction of infarct volume, with the aim to provide reliable target validation data. A priori sample size calculation showed that at least 10 animals would be necessary in each study arm, which we exceeded with n = 41 WT and n = 51 NOX2 KO mice. Importantly, 24 h after transient middle cerebral artery occlusion (tMCAO), neither infarct distribution (Fig. 4A), infarct size (Fig. 4B), nor neurofunctional parameters such as the Bederson score (Fig. 4C) or the Grip test (Fig. 4D) were significantly different between NOX2 KO (n = 51) and WT (n = 41) mice. If one was to examine the possible difference in infarct size of −10% in NOX2 (for which our study was not powered), a much larger study with n = 202 animals per study arm would be needed (based on a power of 1 − β = 0.08, and an α of 0, 05, a standard deviation of 30%, an effect of 10%, and an average reported acute mortality rate of 30%). The use of 404 animals to clarify such a small and translationally insignificant effect would be ethically non-justifiable.

With respect to stroke and NOX, gender-specific effects have been reported^66^. In our study, a sub-group analysis showed in male NOX2 KO mice a larger than average infarct size reduction of 25% (see Fig. 4B, second data set), which reached significance (p = 0.04) but was underpowered (1 − β = 0.68). In none of the subgroups did we find any translationally relevant improvements, neither in neurological behavior or motor function (see Fig. 4C,D).

### Our revised meta-analysis suggests an even lower effect size, no neurological improvement and persisting publication bias

**Figure 5:**
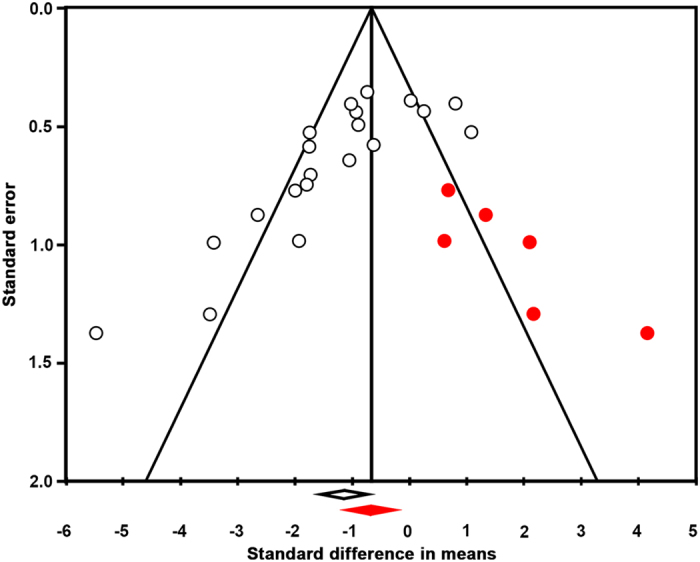
Updated funnel plot with the data from the current trial. Funnel plot asymmetry still suggests the presence of publication bias and an overestimation of the overall effect size of NOX2 in stroke. Using the trim and fill analyses, the intervention effect is adjusted for possible missing studies (filled red symbols) amongst published and present data (open symbols). Studies showing larger infarcts in NOX2 KO mice and thus a smaller or no effect of NOX2 in experimental stroke, are still missing. For further explanations see Fig. 3.

To test whether these new findings would affect our above meta-analysis (see Fig. 2, left data set) we re-ran the extended data set (see Fig. 2, right data set). Still, NOX2 appeared to significantly decrease the infarct size (Fig. 2a SMD −1.15 [−1.67; −0.63]; n = 20; p = 0.000). However, the effect was now smaller and even after including our new study results, a publication bias still appeared to overestimate our overall effect estimation (Fig. 5). Importantly and independently of infarct size, the effect of NOX2 KO on the neurological score was no longer significant (SMD −0.37 [−0.79; 0.06]; n = 12; p = 0.094). Thus even if a small effect on the surrogate infarct size would ever be shown with sufficient power, we can predict it will not translate into any significant neurological outcome improvement. Clearly, this conclusion is a definitive counter-argument against any further clinical development of this target.

## Discussion

Here we provide a feasible solution to a major problem of current biomedical research, the irreproducibility of pre-clinical results leading subsequently to translational failures at the path to the clinic. We show that adopting our SR-MA-pRCT-MA approach to a more large-scale, collaborative way of research will improve research quality and enhance the validity of pre-clinical decision-making on therapeutic targets. Undoubtedly, it also challenges current funding and career reward systems which value rather individual than team achievements. We chose to examine stroke as an example as this is an area of extreme medical need with probably one of the lowest translational success rates in biomedicine. However, we feel confident that what we show applies most likely to many other fields and many other claimed pharmacological targets.

A profound body of pre-clinical literature seemed to suggest that NOX2 is a therapeutic target in stroke whilst some data had argued clearly against this. We hypothesized that this may be a case of insufficient pre-clinical validity on either side, which would qualify to conduct a pRCT. After contacting every group that had published in this field on this pharmacological target we conducted a systematic review. Indeed our subsequent meta-analysis suggested that a small effect of NOX2 on infarct size and neurological score might exist but that there was also a significant publication bias. In the present case, complete reporting of data would have shifted the true effect size towards a lower or no role of NOX2 in stroke. Reporting of essential experimental parameters was also rather poor. Failure in reporting these details is known to skew the interpretation of study results and subsequent translation into clinical benefits. Another criterion that was fulfilled in all studies that reported significant effects was the lack of statistical power to detect a relevant infarct size reduction of 40%^62^, which is a soft target as this will still not ensure clinical benefit^67^,^68^,^69^,^70^. The true threshold is likely to be even higher, but in the absence of any successful translation of pre-clinical stroke research in the past 20 years^14^, this cannot be determined^21^.

Having established that the literature on NOX2 in stroke fulfilled all typical criteria for insufficient pre-clinical target validation (publication bias, poor methodological quality and lack of power), we here present the first SR-MA-pRCT-MA approach to validate a single intervention or, in this case, target validation. Based on its outcome data, a NOX2 KO does not improve neurological outcome and has an effect on infarct size that was too small to be determined by a trial powered for 40% reduction. Conducting another pRCT using >400 animals would be required to determine whether indeed a 10% infarct size reduction occurs; this however would be considered translationally irrelevant (see above examples and threshold) and thus unethical.

Moving to the SR-MA-pRCT-MA approach as the new quality standard, at least for pharmacological target validation, will in all likelihood exceed individual laboratories’ capacity. Thus conducting such studies in a more collaborative manner, meaning multi-center trials, seems to be the logical way forward. In fact, for other targets, including very late antigen 4 (VLA-4)^71^ and transient receptor potential cation channel, subfamily M, member 2 (TRPM2; PMID: 25236871)^72^, such trials are currently under way already. A website has been launched (www.p3pt.de) as an invitation to the community to provide their position on pRCTs and potential suggestions how those should be organized and performed^21^.

Importantly, implications reach further. The studies that we have analyzed were conducted in Korea, Germany, USA, Australia, and the Netherlands. Animal ethics regulations differ, but at least for the European Community it can be said that in recent years there has been a massive push towards ‘The Three Rs’, reduction, replacement, and refinement^73^. However, reducing the number of animals below the limits of statistical power will lead to underpowered and in the end meaningless pre-clinical data sets. Whilst we strongly support the goal to achieve pre-clinical evidence with the least amount of animal sacrifice, the use and reporting of a power calculation is essential to the proper conduct of confirmatory animal studies. Other study formats, e.g. in earlier pre-clinical stages are important as well; however, they should describe themselves as exploratory and not make statements on target validity.

Finally, funding agencies and journals may need to adapt. Funding and career incentives typically reward individuals, whereas pRCTs require team approaches. Journals will need to equally accept for publication well-conducted (e.g. statistically powered) negative findings, so that the literature is truly representative of the science. To ensure that such findings are accessible, even if not submitted for publication, a rather far-reaching but effective measure would be to require pre-registration of animal experiments, whether they were conducted as pRCTs or just pilots, similar to requirements for clinical trials. Registration would be a pre-requisite for ethics approval and include the obligation to subsequently enter the data into a publicly available database. This will reduce the efforts to conduct MAs as an interim surrogate for pRCTs. However, conducting fully powered pRCTs, including detailed reporting and subsequent MA is clearly the way forward.

## Additional Information

**How to cite this article**: Kleikers, P.W.M. *et al.* A combined pre-clinical meta-analysis and randomized confirmatory trial approach to improve data validity for therapeutic target validation. *Sci. Rep.* **5**, 13428; doi: 10.1038/srep13428 (2015).

## Supplementary Material

Supplementary Information

## Figures and Tables

**Table 1:** Power analysis of experimental stroke studies validation the role of NOX2 in *Nox2-/y* and *WT* mice.

Reference	Gender	Durationischemia(min)	Time ofsacrifice(h)	Infarct size								Powerfor 40%effect
				NOX2 WT				NOX2 KO				
				N	Mean(mm ^3^)	SD	CV	N	Mean(mm^3^)	SD	CV	
Reperfusion time 24 h, short ischemia												
Brait^38^	M	30	24	8	58,63	32,16	0,55	10	30,04	28,37	0,94	26
	F	30	24	7	24,93	17,04	0,68	10	42,69	17,01	0,40	24
De Silva^35^	M	30	24	15	36,30	13,20	0,36	7	14,70	10,20	0,69	28
Reperfusion time 24 h, middle long ischemia												
Chen^40^	M	75	24	5*	53,90	19,23	0,36	5*	26,00	10,73	0,41	54
Chen^41^	M	75	24	3*	50,00	7,79	0,16	3*	28,50	3,93	0,14	29
Kleinschnitz^24^	M	60	24	10	78,70	19,50	0,25	18	79,18	20,90	0,26	95
McCann^53^	M	60	6	5	12,90	4,30	0,33	5	9,50	2,10	0,22	59
	M	60	24	5	35,60	4,30	0,12	6	15,10	3,10	0,21	54
Reperfusion time 24 h, long ischemia												
Liu^49^	M	90	24	5	38,00†	2,00	0,05	5	25,00†	5,00	0,20	16
Kahles^37^	M	120	24	13	83,88	41,92	0,50	14	43,20	38,23	0,88	41
Tang^36^	M	120	24	6	163,83	35,59	0,22	4	69,41	35,66	0,51	15
Walder^23^	M	120	24	10	54,00	33,52	0,62	13	29,10	20,19	0,69	35
Wang^42^	M	120	24	8	35,25	9,50	0,27	8	19,38	8,55	0,44	25
Reperfusion time 72 h												
Chen^40^	M	75	72	5*	106,20	27,95	0,26	5*	52,00	26,39	0,51	25
Chen^41^	M	75	72	3*	86,00	16,00	0,19	3*	55,00	16,16	0,29	14
Kunz^39^	M	25	72	5*	52,00	24,40	0,47	5*	20,10	11,70	0,58	25
McCann^53^	M	90	72	5	28,77	14,14	0,49	8	20,92	9,28	0,44	31
Reperfusion time 24 h, middle long ischemia, including current study												
Chen^40^	M	75	24	5*	53,90	19,23	0,36	5*	26,00	10,73	0,41	48
Chen^41^	M	75	24	3*	50,00	7,79	0,16	3*	28,50	3,93	0,14	26
Kleinschnitz^24^	M	60	24	10	78,70	19,50	0,25	18	79,18	20,90	0,26	92
McCann^53^	M	90	6	5	12,90	4,30	0,33	5	9,5	2,10	0,22	53
	M	90	24	5	35,60	4,30	0,12	6	15,10	3,10	0,21	48
Multicentre	F	60	24	10	99,77	33,12	0,33	19	75,44	32,99	0,44	97
	M	60	24	21	76,76	10,53	0,14	14	84,66	9,31	0,11	93
	All	60	24	41	89,00	32	0,36	51	80,00	31,00	0,39	100

To assess the power of already published studies, original data sets were requested from the authors. Means, SD’s and numbers of animals were extracted from the original data or, if original data sets could not be obtained, from the text or figures using ‘universal desk top ruler’ software. If only the SEM was reported, the SD was calculated using formula (2).

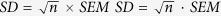
 (2).

For four different groups, pooled variances were used to calculate the power for detecting a 40% difference was calculated using the Russ Lenth’s power and sample size software (http://homepage.stat.uiowa.edu/~rlenth/Power/index.html). The power for the ‘reperfusion time 24 h, middle long ischemia’ group were recalculated using the newly available data from the current pRCT study. * Lowest number of number range † Infarct size reported as percentages.
