# Supplementary material for: A combined pre-clinical meta-analysis and randomized confirmatory trial approach to improve data validity for therapeutic target validation
Source: Sci Rep. 2015 Aug 27;5:13428. doi: 10.1038/srep13428 (PMC4550831; doi:10.1038/srep13428)
Supplement: Supplementary Information [file srep13428-s1.docx]

# SUPPLEMENTARY INFORMATION

**A combined pre-clinical meta-analysis and randomized, confirmatory trial approach to improve data validity for therapeutic target validation**

Pamela WM Kleikers,^1, †^ Carlijn Hooijmans,^2, †^ Eva Göb,^3, †^ Friederike Langhauser,^3^ Sarah SJ Rewell,^4^ Kim Radermacher,^1^ Merel Ritskes-Hoitinga,^2^ David W Howells,^4^ Christoph Kleinschnitz,^3,§^ Harald HHW Schmidt^1,*^

1 Department for Pharmacology, CARIM, Faculty of Health, Medicine and Life Sciences, and Maastricht Institute for Advanced Studies, Maastricht University, The Netherlands, 2 SYRCLE at Central Animal Laboratory, Radboud University Medical Centre, Nijmegen, The Netherlands, 3 Neurologische Klinik und Poliklinik der Universitätsklinik Würzburg, Würzburg, Germany, 4 Florey Institute of Neuroscience and Mental Health, Austin Health, Melbourne, Victoria, Australia.

Correspondence and requests for materials should be addressed to H.H.H.W.S. (email: h.schmidt@maastrichtuniversity.nl)

# SUPPLEMENTARY FIGURES

**Supplementary Figure 1 | Flow diagram showing the different stages of the systematic review and meta-analysis on the possible role of NOX2 in stroke.** Stages include identification (search of papers using Pubmed and Embase), screening (first stage eligibility screening based on title and abstract), eligibility (full text screening, using in- and exclusion criteria), and inclusion (studies included in quantitative and qualitative analysis). Exclusion criteria are listed on the right.


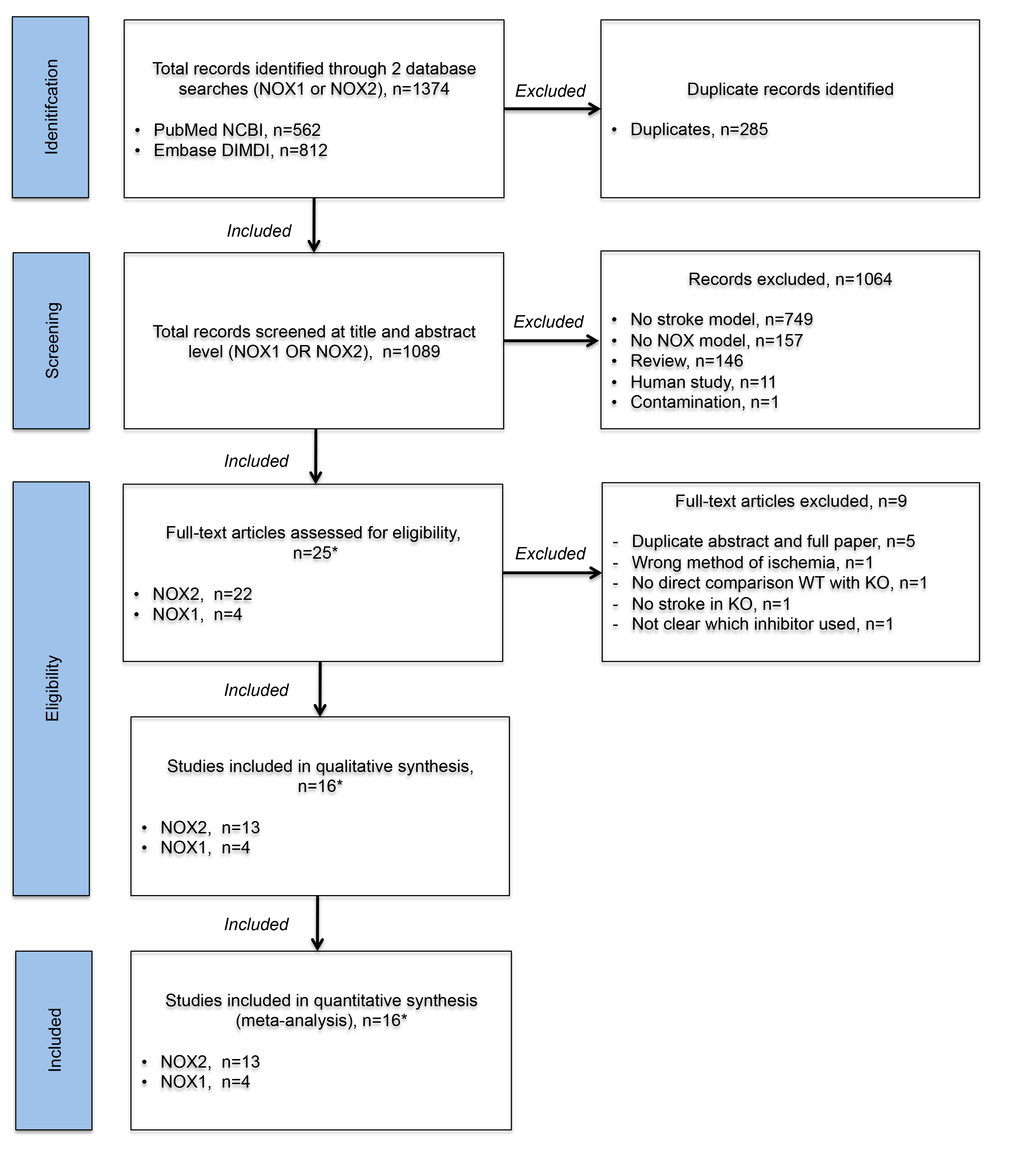


**Supplementary Figure 2 | Risk of bias and reporting quality of NOX1 studies.** For further explanation refer to Fig 1.


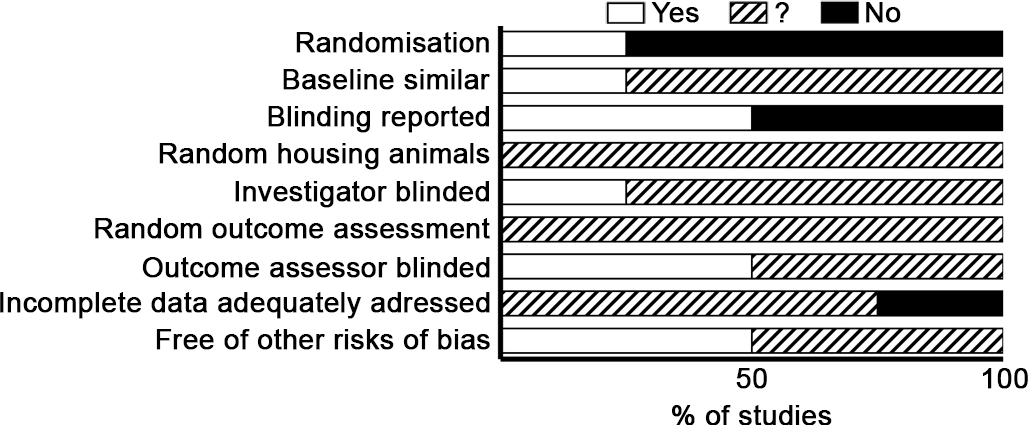


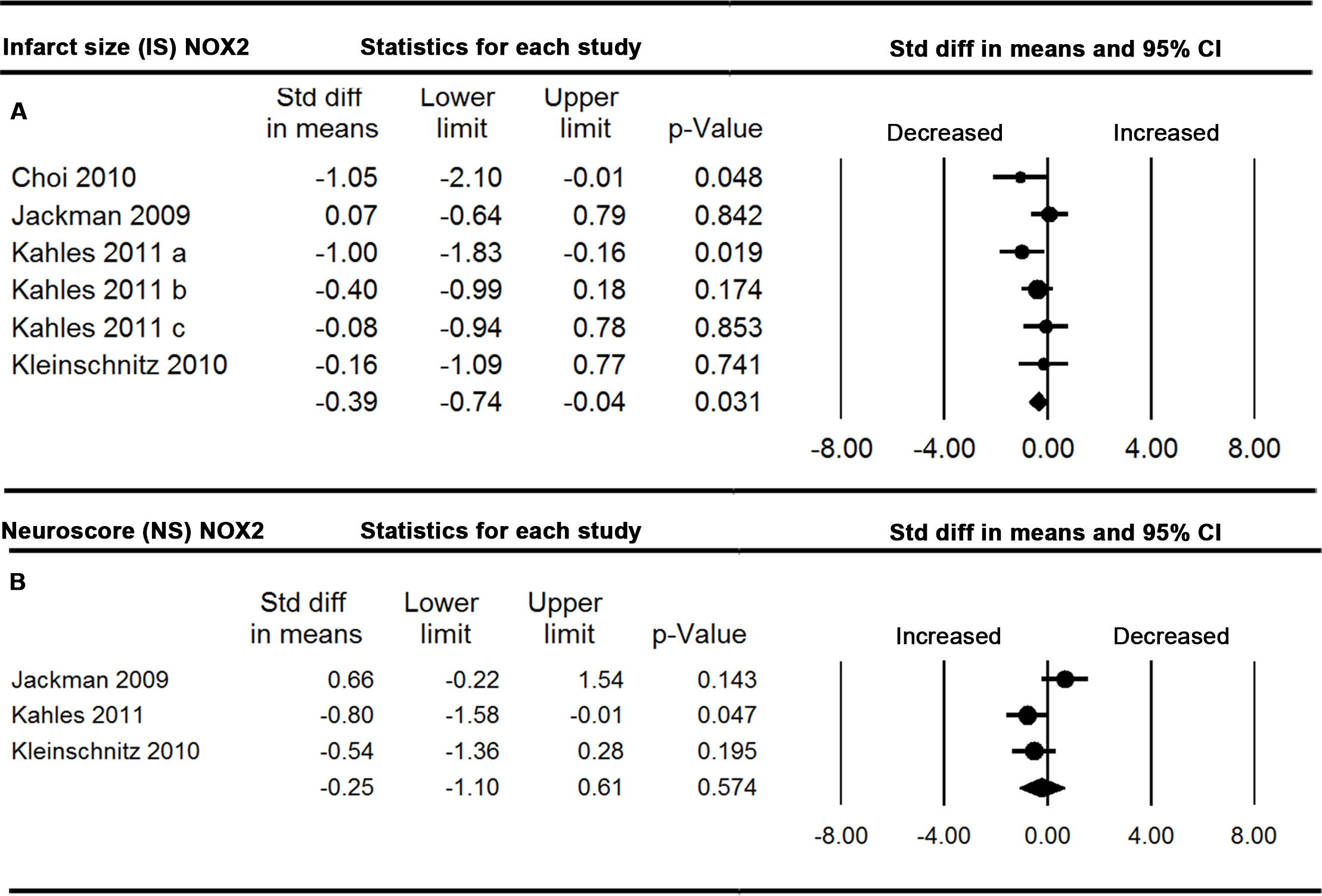
**Supplementary Figure 3** | **Meta-analysis of the overall effect of NOX1 on infarct size and neurological score in experimental stroke.** Studies included are shown on the left and analyzed by Forest plots on the right. Subgroups within one study are depicted separately with the following coding: a, female gender; b, male gender; c, short ischemic time. The upper half (A) contains data for the effect of NOX1 on infarct size (IS); the lower half (B), on neurological score (NS). Displayed are the standardized mean difference (SMD), 95% confidence intervals and relative weight of the individual studies. The diamond indicates the global SMD and its 95% confidence interval. NOX1 has no effect.

# SUPPLEMENTARY TABLES

**Supplementary Table 1: Search strategy systematic review.**

| Database | Search component | Search term |
| --- | --- | --- |
| PubMed | Experimental stroke | ((brain[tiab] OR brains[tiab] OR Cerebrovascular[tiab] OR cerebral[tiab] OR cerebellum[tiab] OR cortical[tiab] OR intracranial[tiab] OR intracerebral[tiab] OR infratentorial[tiab] OR supratentorial[tiab] OR hemisphere OR hemispheric OR MCA[tiab] OR ACA[tiab] OR anterior circulation[tiab] OR posterior circulation[tiab] OR neuron[tiab] OR neuronal[tiab]) AND (ischemia[tiab] OR ischaemia[tiab] OR infarct[tiab] OR infarcts[tiab] OR infarction[tiab] OR infarctions[tiab] OR occlusion[tiab] OR occlusions[tiab] OR obstruction[tiab] OR obstructions[tiab] OR haemorrhage[tiab] OR hemorrhage[tiab] OR haemorrhages[tiab] OR hemorrhages[tiab] OR haematoma[tiab] OR haematomas[tiab] OR hematoma[tiab] OR hematomas[tiab] OR bleeds[tiab] OR bleeding[tiab] or bleeding[tiab] OR bleed[tiab] OR stenose[tiab] OR stenoses[tiab])) OR ((lacunar[tiab] OR cortical[tiab]) AND (infarct[tiab] OR infarcts[tiab] OR infarction[tiab] OR infarctions[tiab])) OR ((ACA[tiab] OR MCA[tiab] OR middle cerebral artery[tiab] OR anterior cerebral artery[tiab] OR Posterior Cerebral Artery[tiab]) AND ( infarct[tiab] OR infarcts[tiab] OR infarction[tiab] OR infarctions[tiab] OR occlusion[tiab] OR occlusions[tiab] OR obstruction[tiab] OR obstructions[tiab])) OR Stroke[tiab] OR Strokes[tiab] OR Vascular Accident[tiab] OR Cerebrovascular Accident[tiab] OR Cerebrovascular Accidents[tiab] OR CVA[tiab] OR Venous Infarction[tiab] OR Anterior Cerebral Circulation Infarction[tiab] OR MCAO[tiab] OR Apoplexy[tiab] OR hemisphericstroke[tiab] OR ("Intracranial Hemorrhages"[Mesh]) OR "Stroke"[Mesh] OR "Brain Infarction"[Mesh] OR "I/R"[Tiab] OR "IRI"[Tiab] OR "Ischemic reperfusion injury"[Tiab] OR "Ischaemic reperfusion injury"[Tiab] OR "Ischemic reperfusion injuries"[Tiab] OR "cerebral Ischaemic reperfusion "[Tiab] OR "cerebral Ischemic reperfusion"[Tiab] OR "reperfusion injury"[tiab] OR "reperfusion injuries"[tiab] OR "ischemia reperfusion"[tiab]OR "ischaemia reperfusion"[tiab] |
|  | NADPH oxidase 1/2 | Nox [tiab] OR NOX 1 [tiab] OR Nox1 [tiab] OR Nox-1 [tiab] OR NOX 2 [tiab] OR Nox2 [tiab] OR Nox-2 [tiab] OR NADPH Oxidase [tiab] OR NADPH oxidases [tiab] OR NADPH-oxidase [tiab] OR NADPH-oxidases [tiab] OR NAD(P)H oxidase [tiab] OR NAD(P)H oxidases [tiab] OR NAD(P)H-oxidase [tiab] OR NAD(P)H-oxidases [tiab] OR NOX1/NADPH [tiab] OR NOX2/NADPH [tiab] OR OR NADPH oxidase-derived superoxide [tiab] OR NADPH oxidase-deficient mice [tiab] glycoprotein gp 91 phox[tiab] OR glycoprotein gp91 phox[tiab] OR glycoprotein gp91phox[tiab] OR gp 91 phox[tiab] OR gp91phox[tiab] OR Gp91phox [tiab] OR gp91-phox [tiab] OR gp91(phox) [tiab] OR NOX1/NADPH [tiab] OR "NADPH Oxidase"[Mesh] OR "NADH, NADPH Oxidoreductases"[Mesh] OR "NADPH oxidase 1" [Supplementary Concept] |
|  | Animal | Search filter for animal studies Pubmed ^44^ |
| *Supplementary table 1, continued* | | |
| EMBASE | Experimental stroke | ((brain.ti,ab. OR brains.ti,ab. OR Cerebrovascular.ti,ab. OR cerebral.ti,ab. OR cerebellum.ti,ab. OR cortical.ti,ab. OR intracranial.ti,ab. OR intracerebral.ti,ab. OR infratentorial.ti,ab. OR supratentorial.ti,ab. OR hemisphere.ti,ab. OR hemispheric.ti,ab. OR MCA.ti,ab. OR ACA.ti,ab. OR anterior circulation.ti,ab. OR posterior circulation.ti,ab. OR neuron.ti,ab. OR neuronal.ti,ab.) AND (ischemia.ti,ab. OR ischaemia.ti,ab. OR infarct.ti,ab. OR infarcts.ti,ab. OR infarction.ti,ab. OR infarctions.ti,ab. OR occlusion.ti,ab. OR occlusions.ti,ab. OR obstruction.ti,ab. OR obstructions.ti,ab. OR stenose.ti,ab. OR stenoses.ti,ab.)) OR ((lacunar.ti,ab. OR cortical.ti,ab.) AND (infarct.ti,ab. OR infarcts.ti,ab. OR infarction.ti,ab. OR infarctions.ti,ab.)) OR ((ACA.ti,ab. OR MCA.ti,ab. OR middle cerebral artery.ti,ab. OR anterior cerebral artery.ti,ab. OR Posterior Cerebral Artery.ti,ab.) AND ( infarct.ti,ab. OR infarcts.ti,ab. OR infarction.ti,ab. OR infarctions.ti,ab. OR occlusion.ti,ab. OR occlusions.ti,ab. OR obstruction.ti,ab. OR obstructions.ti,ab.)) OR Stroke.ti,ab. OR Strokes.ti,ab. OR Vascular Accident.ti,ab. OR Cerebrovascular Accident.ti,ab. OR Cerebrovascular Accidents.ti,ab. OR CVA.ti,ab. OR Venous Infarction.ti,ab. OR Anterior Cerebral Circulation Infarction.ti,ab. OR MCAO.ti,ab. OR Apoplexy.ti,ab. OR hemisphericstroke.ti,ab. OR exp brain ischemia/ OR exp stroke/ OR exp brain infarction/ OR exp brain hemorrhage/ OR "I/R".ti,ab. OR "IRI".ti,ab. OR "Ischemic reperfusion injury".ti,ab. OR "Ischaemic reperfusion injury".ti,ab. OR "Ischemic reperfusion injuries".ti,ab. OR "cerebral Ischaemic reperfusion ".ti,ab. OR "cerebral Ischemic reperfusion".ti,ab. OR "reperfusion injury".ti,ab. OR "reperfusion injuries".ti,ab. OR "ischemia reperfusion".ti,ab.OR "ischaemia reperfusion".ti,ab. |
|  | NADPH oxidase 1/2 | Nox.ti,ab. or NOX 1.ti,ab. or Nox1.ti,ab. or Nox-1.ti,ab. or NOX 2.ti,ab. or Nox2.ti,ab. or Nox-2.ti,ab. or NADPH Oxidase.ti,ab. or NADPH oxidases.ti,ab. or NADPH-oxidase.ti,ab. or NADPH-oxidases.ti,ab. or NAD P H oxidase.ti,ab. or NAD P H oxidases.ti,ab. or NOX1 NADPH.ti,ab. or NOX2 NADPH.ti,ab. or NADPH oxidase-derived superoxide.ti,ab. or NADPH oxidase-deficient mice.ti,ab. or Gp91phox.ti,ab. or glycoprotein gp 91 phox.ti,ab. or glycoprotein gp91 phox.ti,ab. or glycoprotein gp91phox.ti,ab. or gp 91 phox.ti,ab. or gp91phox.ti,ab. or gp91-phox.ti,ab. or gp91 phox.ti,ab. or exp reduced nicotinamide adenine dinucleotide phosphate oxidase/ or exp reduced nicotinamide adenine dinucleotide phosphate oxidase 1/ or exp reduced nicotinamide adenine dinucleotide phosphate oxidase 2 |
|  | Animal | Search filter for animal studies in Embase ^45^ |

**Supplementary Table 2: Characteristics of the studies included in the meta-analysis.**

| Study | |  | Animal characteristics | | | | | |  | Study design | | | |  | Outcome assessment | | | | | | |  |
| --- | --- | --- | --- | --- | --- | --- | --- | --- | --- | --- | --- | --- | --- | --- | --- | --- | --- | --- | --- | --- | --- | --- |
|  |  |  | Species | Strain | Control group | Gender | Age (wks) | Weight (gram) |  | Filament* | t/pMCAO | Duration ischemia | Anaesthetics |  | Timing vs ischemia (h) | Method of culling | Infarct size method | Neurological scoring | | Mortality | |  |
| NOX1 | Choi ^52^ |  | R | SD | n.a | M | 8 | 275-300 |  | 4-0 | T | 90 | Isoflurane 1.75% |  | 24 | Perfusion formalin | Cresyl violet | ? | | ? | |  |
|  | Jackman ^50^ |  | M | NOX1 KO | NOX1 WT | M | 11-17 | 26-28 |  | 6-0 | T | 30 | Ketamine Xylazine† |  | 24 | Inhalation CO_2_ + deca-  pitation | Thionin | Simplified Bederson | | ? | |  |
|  | Kahles ^51^ |  | M | NOX1 KO | C57Bl6/N | M | ? | ? |  | 8-0 | T  P | 60  120 | Isoflurane 1.5% |  | 24 | ? | TTC | 5-point score | | WT 15% KO 44% | |  |
|  | Klein  schnitz ^24^ |  | M | NOX1 KO | C57Bl6/N | M | 6-8 | 20-25 |  | 6-0 | T | 60 | Enflurane |  | 24 | ? | TTC | Bederson+grip test | | ? | |  |
| NOX2 | Brait ^38^ |  | M | NOX2KO | C57Bl6J | M  F | 6-8 | 17,5-23 |  | 6-0 | T | 30 | Ketamine Xylazine† |  | 24 | Inhalation CO2 | Thionin | Hanging wire | | WT 13% KO 0%  WT 14% KO 0% | |  |
|  | Chen ^40^ |  | M | Gp91phox KO | WT | M | ? | 25-30 |  | 6-0 | T | 75 | Isoflurane 1,0% |  | 72  24 | Isoflurane+ decapitation | TTC | Modified 5-point score | | ? | |  |
|  | Chen ^41^ |  | M | Gp91phox KO | WT | M | 12-16 | 25-30 |  | 6-0 | T | 75 | Isoflurane 1,0% |  | 72  24 | Isoflurane+ decapitation | TTC | /// | | ? | |  |
|  | Kahles ^37^ |  | M | Gp91phox KO | C57Bl6 | M | 7-9 | ? |  | 8-0 | T | 120 | Isoflurane |  | 24 | ? | TTC | /// | | ? | |  |
|  | Kim ^48^ |  | M | NOX2KO | C57Bl6 | M | 8-12 | 25-26 |  | 6-0 | P |  | Ketamine Xylazine† |  | 24 | Isoflurane+ decapitation | Thionin | 5-point+  hanging wire | | WT 23%, KO 21% | |  |
|  | Klein  schnitz ^24^ |  | M | NOX2 KO | C57Bl6N | M | 6-8 | 20-25 |  | 6-0 | T | 60 | Enflurane |  | 24 | ? | TTC | Bederson+grip test | | ? | |  |
|  | Kunz ^39^ |  | M | Gp91phox KO | C57Bl6 | M | ? | 20-22 |  | 6-0 ** | T | 25 | Isoflurane 2-2,5% |  | 24 | ? | Cresyl violet | | /// | | ? | |
|  | Liu ^49^ |  | M | Gp91phox | C57Bl6 | M | 7-9 | ? |  | ? | T | 90 | Isoflurane |  | 24 | ? | TTC | | /// | | ? | |
|  | McCann ^53^ |  | M | NOX2KO | NOX2 WT | M | ? | ? |  | ? | T | 60 | ? |  | 24  72 | ? | ? | | /// | | ? | |
|  | de Silva ^35^ |  | M | NOX2 KO | C57Bl6J | M | 8-12 | 25-28 |  | 6-0 | T | 30 | Ketamine Xylazine† |  | 24 | Isoflurane+ decapitation | Thionin | | Hanging wire | | WT 15%, KO 4% | |
|  | Tang ^36^ |  | M | NOX2KO | C57Bl6J | M | ? | 25-30 |  | ? | T | 120 | Isoflurane |  | 24 | Isoflurane+ decapitation | HE | | Modified Bederson | | 20-25% | |
|  | Walder ^23^ |  | M | X-CGD mutant | C57Bl6 | M | 8-10 | 22-23 |  | 5-0 †† | T | 120 | Halothane |  | 24 | ? | ? | | /// | | ? | |
|  | Wang ^42^ |  | M | NOX2 KO | WT | M | 12 | ? |  | ? | T | 120 | ? |  | 24 | Anesthesia+decapitation | TTC | | 5-tiered score | | ? | |

* All filaments were nylon and silicone coated unless stated otherwise † Ketamine and xylazine were used in concentrations of 80mg/kg and 10mg/kg respectively in all studies ** heat blunted †† tip flamed

**Supplementary table 3: Items used for risk of bias assessment.**

| Item no | Item description | Sort of bias assessed |
| --- | --- | --- |
| 1 | Was it reported that the experiments were randomised at any level? | Selection bias (reporting) |
| 2 | Were the groups similar at baseline or was adjusted for confounders in the analysis? | Selection bias |
| 3 | Was it reported that the study was blinded at any level? | Selection bias (reporting) |
| 4 | Were the animals randomly housed during the experiment? | Selection bias |
| 5 | Were the investigators during the course of the experiment adequately blinded from knowledge of which intervention each animal received? | Peformance bias |
| 6 | Were animals selected at random for the outcome assessment? | Detection bias |
| 7 | Were incomplete study data adequately addressed?* | Detection bias |
| 8 | Was the study apparently free of other problems that could pose a high risk of bias?† | Attrition bias |

* Adequate if: all animals included or reasons for missing unlikely related to outcome, missing numbers and reasons equal across groups, imputed using appropriate methods † Other problems that were taken into account as being a high risk of bias: inappropriate influence of funders, inappropriate statistics, design-specific risk of bias, replacement of dropouts, contamination (pooling of drugs), temperature not kept constant during

during surgery.

# SUPPLEMENTARY RESULTS

**Systematic review and meta-analysis on NOX1 in stroke.** Risk of bias analysis showed the same poor reporting as with NOX2 studies (Supplementary Fig. 3). An overall effect on the infarct size, favoring deletion of NOX1 was found when including all studies (Supplementary Fig 3a; SMD -0.39 [-0.74;-0.04]; n=4; p=0.031). This effect is very small and is thus not considered to be of clinical relevance. In addition, the number of studies is very small, making it difficult to interpret these results. Sensitivity analysis showed that deleting the study in which rats were used, abolished the found effect. Thus, when only comparing NOX1 KO vs. NOX1 WT mice, no effect on infarct size was found (SMD -0.31 [-0.66;0.03]; n=3; p=0.075). Heterogeneity between the mice studies was low (I^2^=5%). Neurological scorings were also not found to be different between NOX1 KO and NOX1 WT mice (Supplementary Fig 3b SMD -0.25 [-1.10;0.61]; n=3, p=0.574).

# SUPPLEMENTARY METHODS

**Stroke surgery (tMCAO model).** The model has previously been established by using cerebral blood flow monitoring above the territory of the middle cerebral artery (MCA) in order to visualize a drop of 70-80% in blood flow after successful vessel occlusion by Laser Doppler (Moor Instruments Ltd., Devon, UK). After administration of a painkiller (buprenorphine s.c. 0.1 mg/kg, repeated every 12 hours), animals were anesthetized with isoflurane (induction 5% in air, maintenance 1.5-2.5% in air). Anesthesia was maintained by spontaneous ventilation of isoflurane. The animal was placed on a heating-pad (UNO Roestvaststaal BV, Zevenaar, NL) and rectal temperature was maintained at 37.0°C using a feedback-controlled infrared lamp. Focal cerebral ischemia was induced using an intraluminal filament technique. Using a surgical microscope (Wild M5A, Wild Heerbrugg, Gais, CH), a midline neck incision was made and the right common and external carotid arteries were isolated and permanently ligated. A microvascular clip was temporarily placed on the internal carotid artery. A silicon-coated nylon monofilament (size 6-0, Doccol Corporation, Redlands, CA, USA) was inserted through a small incision into the common carotid artery and advanced into the internal carotid artery until a resistance is felt. The tip of the monofilament should be located intracranially at the origin of the right middle cerebral artery and thereby interrupting blood flow. The filament was held in place by a tourniquet suture that has been prepared before to prevent dislocation during the ischemia period and the wound was closed. Reperfusion was initiated 1 h after occlusion by monofilament removal. After the surgery, wounds were carefully sutured and animals were allowed to recover from surgery in a temperature-controlled cupboard. No animal dropouts occurred during surgery. In total, we conducted surgery on 58 NOX2 KO and 48 C57Bl/6 control animals at two study sites. The success rate of the tMCAO procedure was high, with only one NOX2 KO and 5 WT animal producing no neurological or histological signs of brain damage after the surgery, which were subsequently excluded from further analysis. Acute mortality was low (10% in NOX2 KO, n=6; 4% in WT, n=2) and intrinsic to the stroke pathology (as verified by autopsy).

**Neurological behavior.** The mice were assessed for neurological behavior just before sacrifice to determine the final functional status of the animal. Neurological deficits of the mice that had undergone stroke surgery were measured in a blinded manner on a 0 to 5 scale by using the Bederson Score ^73^ with the following definitions: Score 0, no apparent neurological deficits; 1, body torsion and forelimb flexion; 2, right side weakness and thus decreased resistance to lateral push; 3, unidirectional circling behavior; 4, longitudinal spinning; 5, no movement.

**Motor function.** Prior to sacrifice, the mice were also scored for neurological motor deficit according to the Grip Test ^74^. Each mouse was given a discrete value from 0 to 5. This score is used to evaluate motor function and coordination. The apparatus is a metallic rod (0.22 cm diameters, 50cm length) between two vertical supports at a height of 40 cm over a flat surface. The animal is placed mid-way on this rod and is rated according to the following system: Score 0, falls off; 1, hangs on to string by one or both fore paws; 2, as for 1, and attempts to climb on to string; 3, hangs on to string by one or both fore paws plus one or both hind paws; 4, hangs on to string by fore and hind paws plus tail wrapped around string; 5, escape (towards the supports).

**Infarct Volume Measurements.** The ischemic lesion was measured 24 hours after MCAO. The mice were sacrificed by decapitation. The brain was carefully dissected out, freed from the dura mater and placed in a coronal mouse brain slicer (Zivic Instruments, Pittsburgh, PA, USA). Three sequential, 2 mm-thick coronary slices of the brain were cut with razor blades directed by the brain matrix. The slices were then soaked for 10 min in a freshly-prepared solution of 2% 2,3,5- triphenyl tetrazolium hydrochloride (TTC, Sigma-Aldrich, Zwijndrecht, NL) in PBS (pH 7.4) in a small Petri dish, maintained at 37°C in a heater. Excess TTC was then drained, and the slices were washed with PBS and then photographed under the microscope (Dino-Lite Microscope Eyepiece Camera AM 423X). Each brain section was photographed with a ruler. The digital photographs of the TTC stained brain sections were imported into an image analysis program using the software “Leica QWin Pro” for infarct volume measurement. The program quantitatively measures (in mm2) the areas of the infarcted region, the areas of the left and right hemisphere and the total area of the brain slice. Total infarct volume was calculated by adding up infarct volumes of the different sections: V_infarct_ section 1 (mm^3^) = infarct area section 1 (mm^2^) x slice thickness (2 mm). Infarct volumes are corrected for brain edema according to the following equation V_corrected_ (mm^3^) = V_infarct_ x (1-(V_i_-V_c_)/V_c_ with V_i_-V_c_ representing the volume difference between the ipsilateral (V_i_) and contralateral (V_c_) hemisphere and (V_i_-V_c_)/V_c_ expressing this difference as % of the control hemisphere. Brain edema volume was calculated by subtracting corrected from uncorrected infarct volumes ^75^

**SUPPLEMENTARY REFERENCES**

73. Bederson, J. B. *et al.* Rat middle cerebral artery occlusion: evaluation of the model and development of a neurologic examination. *Stroke* **17,** 472–476 (1986).

74. Moran, P. M., Higgins, L. S., Cordell, B. & Moser, P. C. Age-related learning deficits in transgenic mice expressing the 751-amino acid isoform of human beta-amyloid precursor protein. *Proc Natl Acad Sci U S A* **92,** 5341–5345 (1995).

75. Kraft, P. *et al.* Deficiency of vasodilator-stimulated phosphoprotein (VASP) increases blood-brain-barrier damage and edema formation after ischemic stroke in mice. *PLoS ONE* **5,** e15106 (2010).
